# Magnetic Resonance Imaging to Visualize Stroke and Characterize Stroke Recovery: A Review

**DOI:** 10.3389/fneur.2013.00060

**Published:** 2013-05-27

**Authors:** Bradley J. MacIntosh, Simon J. Graham

**Affiliations:** ^1^Physical Sciences Platform, Sunnybrook Research InstituteToronto, ON, Canada; ^2^Heart and Stroke Foundation Centre for Stroke Recovery, Sunnybrook Research InstituteToronto, ON, Canada; ^3^Department of Medical Biophysics, University of TorontoToronto, ON, Canada

**Keywords:** stroke, stroke recovery, magnetic resonance imaging, diffusion, perfusion, functional MRI, arterial spin labeling, review

## Abstract

The global burden of stroke continues to grow. Although stroke prevention strategies (e.g., medications, diet, and exercise) can contribute to risk reduction, options for acute interventions (e.g., thrombolytic therapy for ischemic stroke) are limited to the minority of patients. The remaining patients are often left with profound neurological disabilities that substantially impact quality of life, economic productivity, and increase caregiver burden. In the last decade, however, the future outlook for such patients has been tempered by movement toward the view that the brain is capable of reorganizing after injury. Many now view brain recovery after stroke as an area of scientific research with large potential for therapeutic advances, far into the future (Broderick and William, 2004). As a probe of brain anatomy, function and physiology, magnetic resonance imaging (MRI) is a non-invasive and highly versatile modality that promises to play a particularly important role in such research. Here we provide a basic review of MRI physical principles and applications for assessing stroke, looking toward the future role MRI may play in improving stroke rehabilitation methods and stroke recovery.

## Introduction

Diagnostic imaging is an invaluable aspect of clinical stroke medicine, providing the location, volume, and the nature of the stroke lesion. Anatomical imaging of stroke patients historically has been done with computed tomography (CT), but ever-increasingly is supplanted by the superb soft tissue contrast provided by magnetic resonance imaging (MRI). The versatility of MRI methods also enables much more detailed biophysical information on stroke physiology, above and beyond lesion structure. For example, just after stroke onset, diffusion-weighted MRI (DWI), and perfusion-weighted MRI (PWI) provide information about diffusion of water molecules and microvascular blood flow within brain tissue, respectively. The DWI and PWI methods help to evaluate the ischemic zone surrounding infarcted tissue that is potentially salvageable by recanalization therapies. Magnetic resonance angiography (MRA) approaches are also available to characterize larger-scale vasculature. In the post-acute and chronic phases, functional MRI (fMRI) offers the ability to detect alterations in brain activation patterns post stroke, either associated with a particular behavioral task or during the resting state. It is also possible now to image cerebral blood flow completely non-invasively using arterial spin labeling (ASL) MRI. In this review, we provide biophysical understanding of these and other basic MRI methods, and discuss their application to stroke recovery.

## Basic Physical Principles

Use of MRI in medicine has evolved from the discovery of nuclear magnetic resonance (NMR) in the 1950s (Bloch, 1946; Purcell et al., 1946). The term NMR is apt – *nuclear* refers to specific atomic nuclei which, when placed in a large applied static *magnetic* field, can undergo *resonance* (substantial energy absorption and subsequent energy emission if a pulsed magnetic wave is applied at a certain characteristic frequency). For NMR to occur, the nuclei of interest must have the quantum mechanical property of non-zero “spin.” Such nuclei simplistically can be thought of as very small objects with spinning charge, and therefore exhibiting nuclear magnetic fields. When placed in a large applied magnetic field, the nuclear magnetic fields tend to align with the applied field. The simplest scenario involves the hydrogen nucleus, or proton, which can take on two different energy states: a low-energy “spin-up” state in which the nuclear magnetic field partially aligns to the external field, and a higher-energy “spin-down” state in which the nuclear field aligns partially anti-parallel. The word “partial” refers to the fact that in each state, the alignment is not perfect. Rather, the nuclear field “precesses” around the direction of external field. A good analogy for this effect is a spinning top, the axis of which begins to wobble (precess) about the direction of gravity as the top “winds down.” In the case of NMR, the frequency of precession is known as the Larmor frequency. The Larmor frequency is the resonance frequency of the system, and transitions between energy states are possible if a magnetic pulse is applied at the Larmor frequency. Typically, a pulse in the radiofrequency (RF) portion of the electromagnetic spectrum is required for NMR, similar to the frequencies used in FM radio.

The NMR effect is one remarkable example of how quantum mechanical effects occurring over extremely small distances (approximately 10^−10^ m) can manifest as macroscopic, observable signals. Within a material containing NMR-capable nuclei, the nuclear magnetic fields for the given energy states can be thought of as “adding up,” resulting in a net nuclear magnetization. The material is very slightly magnetized when placed in a large static magnetic field, due to the population imbalance in favor of protons in the more favorable low-energy state. The magnetization is typically small at room temperature because thermal energy is sufficient to promote a small change between the spin energy states. If the external magnetic field is made strong enough, the nuclear magnetization increases to the point that a signal can be easily observed above the noise background of a detector circuit. For this reason, current MRI systems operate using superconductor technology to generate very large static magnetic fields of 1.5–3.0 Tesla, with ultra-high field systems at 7.0 Tesla and beyond under development (1 Tesla is approximately 2 × 10^4^ times the Earth’s magnetic field).

The conventional manner for observing nuclear magnetization involves three steps. First, the sample is placed in a large applied magnetic field, creating magnetization in the parallel or “longitudinal” direction. Second, a magnetic RF pulse is applied at the Larmor frequency, causing energy absorption that perturbs the energy states and consequently the magnetization. The RF pulse has the macroscopic effect of “tipping” the magnetization out of alignment with the applied field, leading to a vector component oriented transverse (orthogonal) to the longitudinal direction and that oscillates at the Larmor frequency. Lastly, the oscillating transverse magnetization component is detected as an NMR signal using a “receiver coil.” In its simplest form, a receiver coil consists of an appropriately oriented loop of wire that records an NMR-related oscillating voltage induced by the transverse magnetization, according to Faraday’s Law of Induction.

Early in the development of NMR, it was recognized that hydrogen exhibits the largest nuclear magnetic field and thus the largest NMR signal of all the elements. This is fortuitous, given that hydrogen in water is by far the most biologically abundant of nuclei. An additional strength is the remarkable versatility of NMR, to probe not only the magnetic field properties of the nucleus, but also molecular interactions. Collectively, these effects provide an unrivaled mechanism to probe the soft tissues of the body in a non-invasive manner.

## Main Sources of NMR Signal Contrast

In the early 1970s, it became increasingly apparent that biological tissues often exhibit markedly different NMR signal characteristics (Damadian, 1971). There are several ways to obtain tissue contrast, i.e., the ability to detect different tissue types based on the associated NMR signal differences. First, the number of protons per unit volume, or proton density, is a factor that weights all NMR signals. Typically the proton density of tissues is rather uniform throughout the body and a moderate source of signal contrast. Much improved signal contrast is obtained based on several NMR parameters that characterize how magnetization tends to recover toward its equilibrium condition after the RF pulse is turned off. The RF pulse tips magnetization out of alignment with the external magnetic field, typically producing vector components in the longitudinal direction and in the transverse direction. Magnetization then immediately starts to return to its equilibrium condition, resulting in recovery of longitudinal magnetization, and decay of transverse magnetization. In simple fluids such as water, both processes are exponential in nature. Recovery of longitudinal magnetization is characterized by the time constant T1, whereas decay of transverse magnetization is characterized by the time constant T2. In tissues, typical T1 values range from approximately 300–2000 ms at 1.5 and 3.0 Tesla, respectively, with a tendency for T1 to increase as the applied magnetic field increases. The analogous values for T2 range from approximately 30–300 ms. Table 1 provides proton density, T1, and T2 values for different brain tissues.

**Table 1:** **List of relaxation times by tissue type and main magnetic field svtrength (Gati et al., 1997; Kruger et al., 2001; Lu et al., 2005; Wu and Wong, 2006)**.

Field strength (T)	Tissue	T1 (ms)	T2 (ms)	T2* (ms)	Proton density
1.5	White matter	510	67	78	0.61
	Gray matter	760	77	69	0.69
	Arterial blood	1441	290	55	0.72
	CSF	2650	280	n.a.	1.0
3.0	White matter	1080	70	50	0.61
	Gray matter	1820	100	50	0.69
	Arterial blood	1932	275	46	0.72
	CSF	3817	1442	n.a.	1.0

Table 1 indicates that T1 and T2 values differ by approximately a factor of 10, and vary for different tissues. The reason for tissue-related differences in relaxation times is multifaceted (Bronskill and Graham, 1993), but several of the key issues can be briefly summarized. Considering T1 relaxation first, the recovery of longitudinal magnetization is primarily influenced by re-population of spin states. When an RF pulse causes increased population of the spin-down state, the subsequent probability of spontaneous decays to the low-energy spin-up state is very low. Transitions occur predominantly due to “stimulated emission,” an effect that requires a source of magnetic field fluctuations primarily at the Larmor frequency. The source of these fluctuations is dynamic proton-proton magnetic field interactions from within water molecules and between neighboring water molecules as they tumble and translate. This relaxation process is influenced by factors such as temperature, viscosity, and the presence of ions, macromolecules, and tissue microstructures such as cell membranes. The effect is additionally complicated by the fact that water molecules typically can diffuse through multiple different relaxation environments within tissues during the T1 timescale. The process of T2 relaxation is additionally influenced by the magnetic fields associated with very slow components of molecular motion, as these perturb the Larmor frequency for individual protons and reduce the coherence of nuclear magnetic fields in the transverse plane. This effect explains why T2 is much shorter than T1 in tissues, and why protons from large macromolecules are typically not observed during MRI. In the latter case, these protons have very slow rates of molecular motion and the resulting magnetic field non-uniformity at the nuclear scale leads to T2 values of approximately 10 μs, too small for observation with conventional MRI technology.

Table 1 also includes one other NMR parameter, known as T2*. Both T2 and T2* parameters reflect decay of transverse magnetization, as measured using different NMR experiments. T2 is measured using a technique known as “spin-echo refocusing” (Carr and Purcell, 1954) that measures the decay of transverse magnetization while compensating for all macroscopic sources of magnetic field inhomogeneity in space (e.g., spatial variation in the applied magnetic field). The T2* parameter is measured in the absence of such compensation, and is typically smaller than the T2 value. The relatively fast T2* decay is due primarily to the fact that biological tissues are highly heterogeneous with the constitutive components often supporting slightly different applied magnetic fields at the microscopic level. These differences arise from spatial variations in magnetic susceptibility, a property of all materials. The result is that tissue will exhibit a range of different Larmor frequencies even if the external main magnetic field is perfectly uniform, reducing the coherence of signal oscillation in the transverse plane and causing more rapid decay of transverse magnetization than is measured by the T2 parameter. Thus, T2* provides an additional mechanism to achieve tissue-specific NMR signal contrast.

## Development of MRI

Early investigations of NMR signals from tissues focused particularly on the ability to detect cancer and distinguish malignant from benign lesions, and provided some of the impetus for developing methods to encode NMR signals spatially in the form of images. Key innovators in these subsequent developments were Lauterbur and Mansfield, who received the Nobel Prize in physiology and medicine in 2003. Over the years, the term “nuclear” has been dropped to avoid any potential misconception that the imaging methodology involves use of ionizing radiation – hence the modern acronym MRI. The first whole-body MRI systems were developed for commercial use in the late 1980s, and since then there has been a steady increase in technical capabilities. Today, multifaceted MRI “protocols” are achieved with approximately 1 mm spatial resolution and exquisite image contrast. Specialized sequences of RF pulses are used to provide images weighted by different MRI signal parameters in examination times of approximately 30 min.

The key technological concept that led to the development of MRI is the “imaging gradient” for spatial encoding. The imaging gradient is an electrical circuit that produces a linear change in magnetic field as a function of position. MRI systems provide magnetic field gradients in the three orthogonal directions *x*, *y*, and *z*. Importantly, although the gradient directions are orthogonal, the orientation of the magnetic field from each gradient is always in the direction of the large external magnetic field. Consequently, a given gradient produces a linear variation in Larmor frequency with position and can be used to encode NMR signals in the gradient direction (Lauterbur, 1973). In comparison with the external magnetic field, gradient fields are much smaller, on the order of 10 milliTesla/meter. They are also designed to be pulsed on and off throughout the imaging procedure, enabling spatial encoding in the *x*, *y*, and *z* directions. It is the pulsing of imaging gradients (leading to vibrational forces within the magnet bore) that is responsible for the characteristic buzzing, knocking, or pinging sounds that occur during MRI scans.

Both “multi-slice” and three-dimensional (3D) MRI require multiple repetitions of RF pulse excitation, data acquisition, and recovery periods to obtain all the necessary spatial encoding information required for image reconstruction. Typically, 3D MRI is advantageous over 2D MRI because isotropic spatial resolution is possible and the data can be straightforwardly reformatted post-acquisition into different image plane orientations (e.g., axial, sagittal, coronal, arbitrary oblique). However, 2D MRI is typically more time-efficient and is widely used on this basis. Clinically, both types of imaging have scan times that usually fall within the 1–10 min range. There is also a class of “fast” MRI techniques capable of providing a fully encoded image slice in approximately 100 ms, or multi-slice datasets in approximately 1–2 s. The most common of these techniques is echo planar imaging (EPI) (Stehling et al., 1991), capable of full spatial encoding within a single RF excitation followed by data acquisition during rapidly varying gradient waveforms. EPI can be used to image time-dependent physiological processes that occur on the timescale of seconds, such as the wash-in of injectable contrast agents. However, fast imaging has costs: reduced signal-to-noise ratio (SNR), reduced spatial resolution, and regions of signal loss and substantial geometric distortion arising from sensitivity to magnetic field uniformity.

## MRI of Cerebrovascular Risk Factors

**Figure 1:**
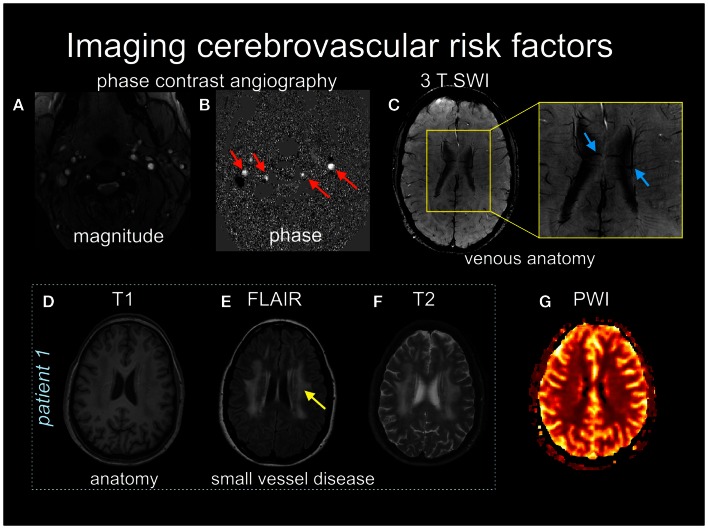
**Multiple MRI pulse sequences can be used to assess cerebrovascular risks**. The magnitude **(A)** and phase **(B)** images in phase contrast angiography can be used to calculate cerebral blood flow velocity in arteries (red arrows) and veins. **(C)** Susceptibility-weighted images (SWI) provide high spatial resolution (<1 mm in plane resolution) of venous anatomy. The inset shows medullary draining veins (blue arrows). **(D–F)** Patient 1 provides structural images, T1 **(D)**, FLAIR **(E)**, and T2-weighted **(F)**, with the latter two images showing subcortical white matter disease (yellow arrow). **(G)** a normal perfusion-weighted image obtained by contrast-enhanced dynamic susceptibility contrast.

Magnetic resonance imaging is being used increasingly as part of the cerebrovascular work-up to assess risk of stroke. Vascular risk factors like hypertension, type 2 diabetes mellitus, and the natural course of aging are associated with an increase in the prevalence of subclinical brain lesions (Thompson and Hakim, 2009). For example, individuals with type 2 diabetes that have brain lesions on MRI appear to have a two to fivefold increase in stroke occurrence compared to non-diabetics (Baird et al., 2002). Cerebrovascular risk factors can manifest as white matter disease (see Figures 1E,F), lacunes, small vessel disease, and/or Virchov Robin spaces, which may be considered subclinical if they do not produce an overt neurological symptom. These features may be viewed as an “incidental finding” but it is more apt to refer to these events as “covert strokes.” For example, meta-analyses show that white matter lesions are a significant risk factor for stroke, dementia, and death (Biernaskie et al., 2004). Thus, as we advance our understanding of the stroke in the acute and chronic stages it is imperative to characterize the underlying cerebrovascular pathologies that may have contributed to the stroke in the first place, and that will also influence the potential for recovery.

## MRI of Acute Stroke

**Figure 2:**
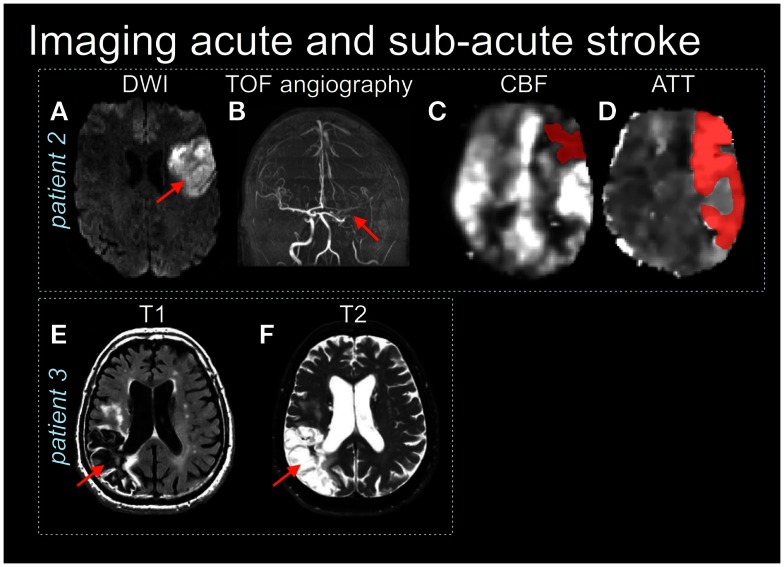
**Patient 2 is an acute stroke with mild impairment (NIHSS = 1) yet the diffusion-weighted image (DWI) (A) shows restricted diffusion in the left frontal lobe (red arrow) and a large vessel obstruction on the × (TOF) angiography (B)**. **(C,D)** Arterial spin labeling images show reduced cerebral blood flow (CBF) and prolonged arterial transit time (ATT) (shaded in dark red) (Based on MacIntosh et al., 2010). Patient 3 shows the right parietal lobe stroke lesion on T1 **(E)** and T2-weighted **(F)** images.

An MRI protocol is a set of individual image acquisitions that each have different signal contrast weightings, assembled with the goal of detecting pathology in a comprehensive manner. Such protocols are becoming increasingly critical in an acute stroke setting, to direct stroke interventions and characterize recovery (Leiva-Salinas et al., 2011). For example, it is possible to design an MRI protocol that identifies regions of possible active ischemia, hemorrhage, and occluded vessels, and that provides insight regarding the size and location of the core infarct as well as the extent of surrounding tissue that potentially can be salvaged (the ischemic penumbra). A typical acute stroke MRI protocol consists of diffusion-weighted imaging (DWI), fluid attenuated inversion recovery (FLAIR), MRA, perfusion-weighted imaging (PWI), and T2-weighted imaging. Some representative examples of these techniques are shown in Figures 1 and 2, as they are sensitive both to overt and covert stroke, with further detail to follow below.

Standard MRI sequences that provide T1-weighting or T2-weighting, and their derivatives, are well known to be insensitive to the immediate effects of cerebral ischemia. By contrast, DWI is highly sensitive to ischemia (Mascalchi et al., 2005). The technique is based on the use of a pair of counter-balanced, pulsed imaging gradients applied in the same direction. The first gradient pulse is used to encode protons as a function of position, whereas the second is used to remove this encoding. If protons are completely static, the counter-balanced gradient pulses have no effect and the resultant MRI signal is proportional to the net magnetization within a voxel. However, if the protons move between the application of the gradient pulses, as is the case for water molecules diffusing in biological tissue, then the spatial encoding is not fully removed and is measured as a signal attenuation providing diffusion-weighted contrast. If the degree of diffusion weighting is manipulated in a series of DWI experiments, simple mathematical modeling enables calculation of an apparent diffusion coefficient (ADC) image, which has information distinct from that provided by proton density or relaxation time parameters.

Within minutes of vessel occlusion, failure of the sodium-potassium pump and associated cytotoxic edema leads to an influx of water from the extracellular space to the intracellular space. This impedes the diffusion of water molecules, as cell membranes act as more of a barrier. The restricted diffusion of water molecules results in increased signal intensity on DWI. The use of DWI is approximately four to five times more sensitive for detecting acute stroke than is non-contrast CT (Chalela et al., 2007).

The combined use of FLAIR and DWI helps to distinguish acute from sub-acute and chronic stroke lesions. The FLAIR technique (De Coene et al., 1992) is a variant of the historic “inversion recovery” NMR experiment, in which an RF pulse is used to invert net magnetization. During the ensuing T1 recovery, the inverted longitudinal magnetization initially shrinks in magnitude, vanishes, and then grows toward its equilibrium value once again. For FLAIR imaging, and timing parameters are adjusted so that the MRI signal is acquired when cerebrospinal fluid is T1-nulled. Given that CSF tends to pool within the infarct zone as time progresses, use of FLAIR combined with DWI can improve the identification of new lesions near sites of prior ischemic injury, potentially providing insight into stroke physiology and subtype. At present there is some debate on whether the FLAIR on its own can be used to predict the time from symptom onset. Typically, FLAIR imaging can detect the presence of ischemia approximately 3 h after stroke (Thomalla et al., 2009). Recent work suggests that using FLAIR relative signal intensity ratios from ischemic to contralateral regions of interest may be better than visual inspection alone for predicting the time of symptom onset. This approach has good sensitivity but poor specificity (Cheng et al., 2013). Time of symptom onset is of critical importance given that the only clinically approved therapy for acute stroke at present, recombinant tissue plasminogen activator (rt-PA), requires administration within several hours of stroke onset (Taussky et al., 2011). Therefore, maximizing the use of rt-PA places an increasing need on the availability and capability of MRI for assessing acute stroke.

Magnetic resonance angiography provides a non-invasive method to screen for vessel occlusions, stenosis, or malformations. There are two MRI techniques available for this purpose, with each performing slightly less sensitively than the gold standard, digital subtraction angiography. Three-dimensional time-of-flight angiography (3DTOF) (Davis et al., 1993) provides a non-invasive method of assessing the intracranial circulation. The technique is based on the fact that if RF pulses are applied rapidly in succession, there is little time for T1 recovery of longitudinal magnetization. Over time, a steady-state of longitudinal magnetization is obtained that is lower than the equilibrium magnetization. However, if blood flows into the imaging plane it still retains its equilibrium value and thus exhibits positive image contrast with respect to the background static tissues. This effect enables imaging of the vascular lumen, but only for through-plane flow. An alternative to 3DTOF is contrast-enhanced magnetic resonance angiography (CE-MRA) (Runge et al., 1993), typically involving the intravenous injection of a paramagnetic contrast agent such as Gadolinium Diethyl-Triamine-Penta-Acetic acid (Gd-DTPA). This agent enhances T1 relaxation, increasing the signal intensity of blood on appropriately T1-weighted images, with respect to the signal from static tissues. This technique provides more contrast than 3DTOF and is much less sensitive to flow dynamics. Consequently, CE-MRA can be used more effectively to image extracranial and intracranial vessels. If scan time permits, the two angiographic methods are complementary and use of both 3DTOF and CE-MRA can improve diagnostic ability (Sohn et al., 2003). Evidence is also accumulating that CE-MRA can be used to scrutinize “beyond the lumen,” improving characterization of vulnerable plaque in the carotid arteries and potentially providing a marker of stroke risk (Wasserman, 2010). A disadvantage of CE-MRA, however, is that Gadolinium contrast agent administration is contraindicated in patients with poor renal function (Morita et al., 2011).

Perfusion-weighted imaging is similar to CE-MRA in that a bolus of Gadolinium contrast agent is administered intravenously, although in this case fast imaging (i.e., EPI or a variant) is employed to generate a time series of images to track reductions in T2*-weighted signal intensity of tissues as the contrast agent travels through the microvasculature. Semi-quantitative perfusion maps are obtainable from this examination that estimate cerebral blood volume (CBV, the blood volume per unit of brain), the mean transit-time (MTT, the average time required by the bolus of contrast agent to cross the capillary network), the cerebral blood flow (CBF, the volume the blood flowing per brain mass and per unit of time, mL/100 g tissue/min). When combined with DWI, both imaging data sets provide information about the ischemic core and about the ischemic penumbra. It was originally thought that the difference between the spatial extent of perfusion deficit and DWI hyperintensity reflected the ischemic penumbra. It is now known that DWI hyperintensity can resolve in many stroke patients (Labeyrie et al., 2012), and that the extent of deficits observed by PWI do not necessarily reflect the true extent of the penumbra. There is some consensus that penumbra imaging can be used in the medical decision making, as in the case of rt-PA therapy (Donnan et al., 2009), however, this imaging approach appears to have generated mixed results in recent endovascular mechanical embolectomy clinical trials (Tenser et al., 2011; Kidwell et al., 2013).

Lastly, T2*-weighted imaging has multiple applications in acute stroke. These applications stem from the fact that the T2* of blood varies with oxygenation content, based on the magnetic susceptibility characteristics of hemoglobin. Oxygenated hemoglobin is diamagnetic, causing a slight decrease in the applied magnetic field that is supported within blood, whereas deoxygenated hemoglobin is paramagnetic, concentrating the magnetic field within red blood cells. Abnormal accumulation of deoxygenated blood thus provides hypointensity on T2*-weighted images and is an indicator of vascular pathology. Thus, T2*-weighted images are capable of detecting acute hemorrhage, with equivalent accuracy to CT (Chalela et al., 2007). Microbleeds, indicative of multiple types of micro-angiopathy are detected on T2*-weighted images (Fazekas et al., 1999), but not on CT due to insufficient signal contrast and spatial resolution. T2*-weighted images are also capable of depicting hemorrhagic transformations of ischemic infarcts, and provide indications for ruling out primary hematoma, as well as depicting thrombosed veins or sinuses. In recent years, T2*-weighted applications are being enhanced through use of a technique known as susceptibility-weighted imaging (SWI; see Figure 1C), which provides improved characterization of susceptibility changes in the brain microvasculature for angiography, venography, and detection of atherosclerosis and thrombosis (Barnes and Haacke, 2009).

## MRI in Stroke Recovery

Despite the continuing technical advances in MRI of acute stroke, these developments represent only the “tip of the iceberg” with respect to health care management of all stroke patients. Currently, only a small fraction of patients are eligible for acute stroke therapy. The remaining stroke survivors are often left with profound deficits, leading to substantial loss in quality of life, economic productivity and substantial caregiver burden (Broderick et al., 1989). In the developed world, the problem is worsening as seniors become more prevalent due to the “baby boom” generation and other factors.

Although MRI is often viewed as not widely accessible and expensive, its versatility and non-invasiveness makes it well suited for studies involving stroke patients to understand stroke pathology and the process of recovery. If useful therapeutic applications arise from such studies, then MRI could be used potentially as a biomarker in the clinic. Used sparingly and appropriately, the costs associated with additional MRI examinations in stroke patients will be small compared to the costs of caregiving, and any substantial reduction in the extent of disability due to stroke introduced by such new therapies will be very important. In the remainder of this review, representative examples are provided that illustrate the breadth of the opportunity, and also the challenges that lie ahead.

## MRI for Neuroanatomical Correlates of Stroke Symptoms

Given the spectrum of deficits exhibited by stroke patients, the variability in location, and extent of ischemic stroke, and the current imperfect understanding of normal brain function in the young and in the elderly, it is not surprising that there are a substantial number of studies that investigate how stroke lesions in specific brain locations correlate with behavioral deficit. The use of stroke patients as a “knock-out” model of brain function can help to clarify the role of a given brain regions, although many strokes are not sufficiently punctate for definitive conclusions and findings must be corroborated through replication and consensus. For stroke patients, improved knowledge of how damaged neuroanatomical structures relate to behavioral deficits can potentially be important for refining cognitive and physical rehabilitation strategies, in the long term.

A simple example illustrating use of MRI in this context involves use of high resolution T1-weighted images to compare the lesion location among chronic ambulatory stroke survivors with symmetric gait compared to patients with asymmetric gait (Alexander et al., 2009). Individuals with asymmetric gait were more likely to have the putamen included in the lesion volume, a structure essential for motor control (see Figure 4).

Such results are increasingly made possible by new developments in image processing that facilitate volumetric analysis of anatomical MRI data. One example is voxel based morphometry (VBM), an approach that involves spatially registering anatomical MRI data from a group to a known “atlas” of brain regions, and then comparing image volumes across brains at each voxel (Ashburner and Friston, 2000). Another approach is measuring the cortical thickness by segmenting cortical gray matter from CSF, and cortical gray matter from subcortical white matter, based on signal intensity differences in high resolution T1-weighted images (Fischl and Dale, 2000). Tissue boundaries can then be represented as convoluted surfaces, with point-to-point thickness estimates obtained by projecting orthogonally from one surface to the other. A recent study used VBM to help classify stroke patients that responded positively to epidural motor cortex stimulation compared to non-responders (Kansagra and Wong, 2008a). One study of cortical thickness found that patients with severe cerebrovascular steno-occlusive disease who underwent surgical revascularization appeared to have replenished the cortex with 5% increase in thickness (Smith et al., 2010). Some of the challenges of cortical thickness methods in cerebrovascular patients relate to the available signal contrast and SNR at 1.5–3.0 Tesla, which may be addressed by ultra-high field MRI (Zwanenburg et al., 2012). Furthermore, misclassification of tissue, advanced atrophy, or in the presence of multiple lesions are additional challenges that may necessitate within-patient analysis approaches that avoid the image processing step of group template coregistration (Longstreth et al., 1998).

### Diffusion tensor imaging

It has been known since the 1990s that there is a preferred direction for water diffusion in certain tissues (i.e., the diffusion is anisotropic) (Henkelman et al., 1994). The effect is pronounced in white matter tracts, with diffusion preferentially occurring along the length of axons, and more restricted in the orthogonal directions. The myelination of axons, the axonal membrane, and microtubules within axons are all thought to contribute to the diffusion anisotropy. If a series of DWI scans are conducted with diffusion weighting in a variety of different orientations, it is possible to estimate the direction and magnitude of diffusion and the correlations between diffusion in different directions, properties that define the anisotropic diffusion tensor and hence lead to the term “diffusion tensor imaging” (DTI) (Nucifora et al., 2007). A minimum of 7 DWI experiments with different diffusion orientations (including one experiment with no diffusion weighting) are required to calculate diffusion tensor parameters, although encoding for more diffusion directions improves data quality. Thus, DTI experiments are often time-consuming (approximately 10 min or longer), even when EPI techniques are used for spatial encoding. The technique can be sensitive to a variety of different image artifacts, including head motion, as the signal attenuation measured in DWI experiments does not differentiate well between movement of water molecules on a microscopic or a macroscopic scale.

**Figure 3:**
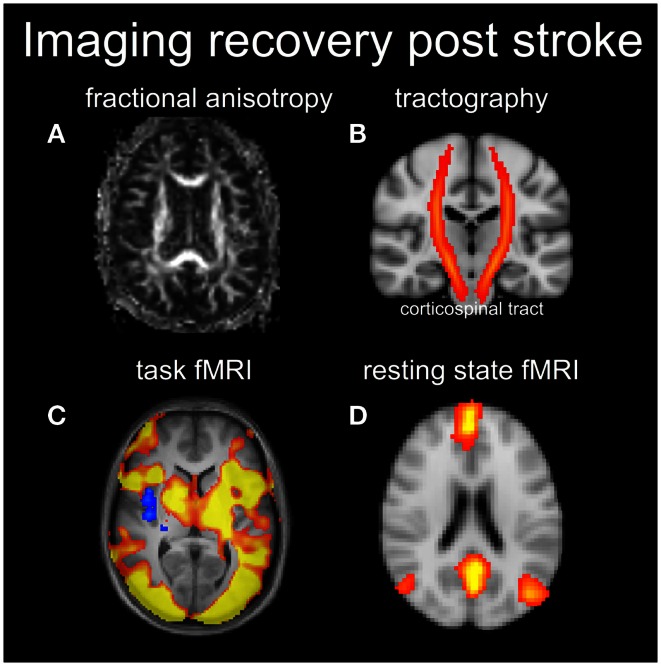
**Diffusion tensor imaging can be used to visualize the fractional anisotropy (FA) (A) in white matter (brighter signal means higher FA) or produce the white matter tracts (B)**. In this case an atlas based corticospinal tract is shown in red and overlaid on a coronal anatomical image. Functional MRI results shows task-based activation **(C)** and a resting-state network corresponding to the default mode network **(D)**.

Data provided by DTI enable calculation of “rotationally invariant” signal contrast parameters that are theoretically independent of which gradient orientations are chosen for diffusion weighting (as long as they are well-spaced in 3D). The mean diffusivity parameter provides contrast similar to that of the ADC, by providing a weighted average of diffusion in three orthogonal directions. A parameter known as the fractional anisotropy (FA) describes the extent that there is a preferred diffusion direction within a particular voxel (shown in Figure 3A). The FA value for gray matter is thus lower than that for white matter, potentially providing a rich source of information relating to brain connectivity.

**Figure 4:**
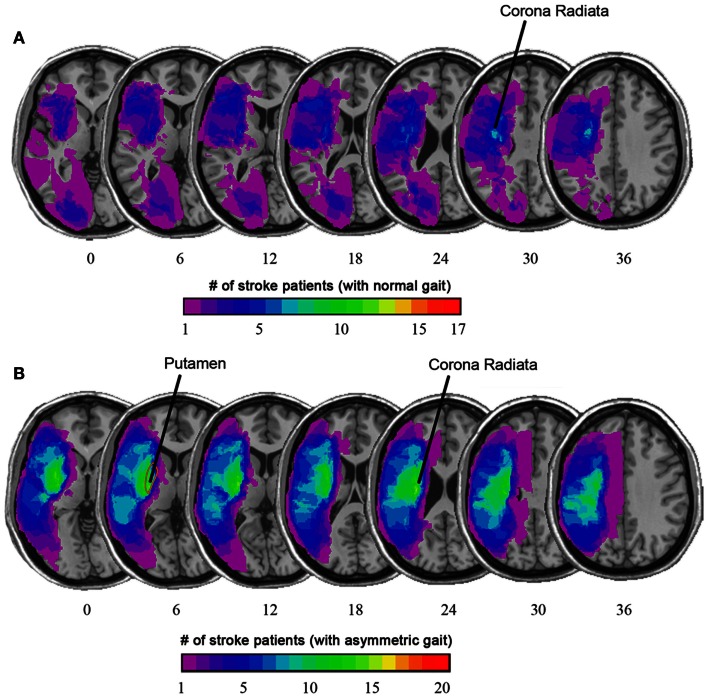
**(A)** Overlaid on the average T1-weighted image is a color map showing the average lesion location as a proportion of stroke patients with normal gait. **(B)** Among stroke patients with asymmetric gait, there was a higher prevalence of stroke lesion that included the putamen and corona radiata. (Based on Alexander et al., 2009).

Location of the stroke lesion will strongly influence the potential for recovery. For example, from lesion analysis work, it is known that a stroke lesion to the internal capsule is particularly problematic for motor recovery because the output of motor signals converges on this subcortical brain structure (Shelton and Reding, 2001). One of the major appeals of DTI for stroke recovery research is therefore the ability to visualize the integrity of the corticospinal tract (shown in Figure 3B). DTI can be used to probe the integrity of the corticospinal tract within the first weeks after stroke, and early DTI measures are highly correlated with residual motor function in the acute and chronic stage (Radlinska et al., 2010). Thus, DTI measures may help to inform whether a patient has the potential to recover a particular behavior or not.

Diffusion tensor imaging data can also be used for tractography, which is a non-invasive method for mapping the paths of white matter fibers in the brain (Ashburner and Friston, 2000). Remarkable pictures of white matter tracts have been generated in monkeys (Adluru et al., 2012) and in the post mortem human brain (Kansagra and Wong, 2008b) although this image quality is currently logistically impractical in routine clinical MRI due to the long scan times that are required. Part of the reason for these long scan times is that the basic diffusion tensor model for white matter does not account well for regions of the brain that have crossing fibers. More sophisticated models require more gradient orientations, and high spatial resolution is paramount. This is a promising methodology for use in stroke patients, and concerted research efforts are under way to improve MRI hardware and enhance the clinical practicality of DTI tractography (Van Essen et al., 2012). For now, however, FA measures derived from DTI remain the primary method of quantifying anisotropic diffusion.

### Functional MRI

Blood oxygenation level-dependent (BOLD) fMRI (Ogawa et al., 1990; Borresen and Lambert, 2008) has revolutionized neuroscience through its capability to measure signal changes associated with neuronal activity generated by sensory stimuli, or by behavioral tasks involving memory, cognition, action, or emotion. Neurons communicate with glial cells and the nearby microvasculature to signal for the delivery of additional blood through vasodilation, when neuronal activity levels increase. The neurovascular unit that governs such processes represents the ultimate spatial resolution achievable with hemodynamic measures of brain activity, in the range of hundreds of microns (Menon and Goodyear, 1999). Due to contrast to noise ratio considerations, however, fMRI studies are typically conducted with voxel dimensions of several millimeters. Researchers have determined that the local field potentials arising from healthy populations of neurons are strongly correlated with BOLD-fMRI signals (Logothetis et al., 2001).

In healthy individuals with an intact neurovascular unit, the BOLD-fMRI response to neuronal activity is characterized by three distinct phases: (1) a fast response lasting 1–2 s when there is a very small decrease in the BOLD signal, (2) a larger hyperemic signal increase caused by the inflow of oxygenated blood, peaking approximately 4–5 s after the neural stimulus, and (3) a refractory period lasting approximately 10 s during which the BOLD signal undershoots and then reaches the baseline level (Norris, 2006). The period of hyperemia provides the most robust means to detect increases in brain activity and the shape of this hemodynamic response is often modeled as a gamma-variate function (Cohen, 1997). BOLD signal characteristics are a complex function of multiple physiological parameters such as CBF, CBV, cerebral rate of metabolic oxygen consumption, and hematocrit. The key property used for generating fMRI, through which these other parameter changes are viewed, is the effect of oxygenation status on the net magnetic susceptibility of blood (oxyhemoglobin is diamagnetic and deoxyhemoglobin is paramagnetic, as described above). Thus, the predominant method for measuring BOLD-fMRI signal changes at 1.5 Tesla and 3.0 Tesla involves using T2*-weighted EPI technique to generate time-series data during periods of neuronal activity. The resulting signal changes are typically small, only a few percent from signal baseline, partly due to the small volumetric fraction of blood in tissues. The upper bound for the BOLD signal, which occurs when deoxyhemoglobin concentrations become negligible, has been estimated as approximately 6% in gray matter at 3T (Bulte et al., 2012). Instances where computed BOLD-fMRI signal changes are larger than 6% should be scrutinized rigorously for spurious signal contamination, from sources such as larger-scale vasculature (Nencka and Rowe, 2007) and head motion. Regarding the latter source of artifact, it is unfortunately the case that head motions of a few millimeters easily contaminate BOLD-fMRI signals. This has long been a problem that affects clinical utility of fMRI, although recent technological improvements are beginning to have an impact (Rotenberg et al., 2013).

### Task-based BOLD fMRI

The majority of the fMRI literature includes task-based experiments, whereby participants alternate between different behavioral or stimulus conditions to induce a measurable BOLD signal change. For example, the simplest approach is to alternate between one specific stimulus/task and a resting condition (often visual fixation on a displayed cursor). When the stimulus/task is very brief (approximately 0.1–4 s), the experimental design is described as “event-related,” whereas longer duration task and control condition periods (typically 15–30 s) are used in “block-designs” (shown in Figure 3C). In stroke recovery research, the majority of fMRI studies have used a block-design primarily because the block-design affords greater detection sensitivity (Calautti and Baron, 2003). Irrespective of the experimental design, the task of interest is undertaken in multiple repetitions to improve the ability to detect brain activity under limited SNR conditions. The majority of fMRI analyses use a simple General Linear Model approach to determine which voxels in the brain show signal patterns consistent with the task design, while accounting for the sluggishness of the BOLD hemodynamic response (Price et al., 2006).

The first task-based fMRI experiment involving stroke patients dates back to 1997, in which Cramer and colleagues (Cramer et al., 1997) visualized the brain regions involved when stroke survivors moved their affected limb. Since this pioneering work, there have been numerous seminal fMRI studies that have helped to characterize brain activation patterns that indicate positive or negative outcomes after stroke. For example, one sign of poor recovery is significant activation in the unaffected hemisphere (i.e., contralateral to the side of the lesion) when performing a unilateral task with the affected limb. Another is diffuse activation of brain regions during the execution of a motor task, in regions typically not observed in healthy individuals performing the same behavior. These observations are supported by a long list of evidence in the fMRI literature, reviewed by Calautti and Baron (2003) from cross-sectional and longitudinal fMRI studies.

**Figure 5:**
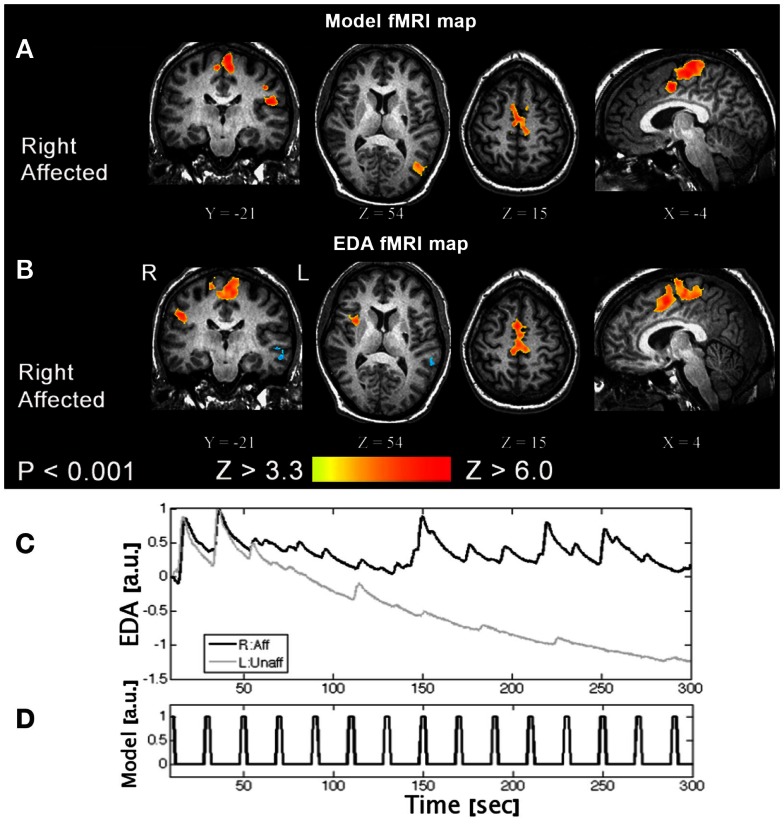
**A task-based fMRI example from a patient with a right side stroke affecting their right leg**. **(A)** An activation pattern generated using a standard fMRI model and **(B)** an activation pattern using an alternative approach, that is the patient’s electrodermal activity time-series data measured concurrently. **(C)** Moving the right affected limb (R:Aff) resulted in larger and repeated phasic EDA responses compared to the left unaffected (L:Unaff) limb, where EDA responses are small and habituate. **(D)** The event-related fMRI paradigm is shown for reference. These results suggest moving the affected limb increased arousal and/or effort (Based on MacIntosh et al., 2008).

One of the challenges of task-based fMRI in stroke recovery research is the issue of task performance. It is important to characterize properly what the patient “does” during fMRI, to rule out the possibility that their activation patterns are different from normal individuals simply because they did the task differently (e.g., speed and extent, applied force, and level of cognitive effort when performing a motor task). One strategy to circumvent this important confound for motor fMRI is to measure relevant biophysical signals concurrently, such as electromyography (EMG) (MacIntosh et al., 2007) or electrodermal activity (EDA) (MacIntosh et al., 2008). EMG provides important information regarding the timing and intensity of muscle contraction, and potentially the muscle groups that are activated, if multichannel recordings are undertaken. EMG may be used to determine if mirror movements confound the interpretation of brain activation patterns, when unilateral movement is prescribed. Alternatively, EDA recordings provide an indirect measure of the autonomic nervous system, probing aspects of arousal, emotion, and sense of effort. EDA recordings have been used to characterize sense of effort during the performance of a motor task with the affected limb post stroke, as represented in Figure 5.

Despite the wealth of information that can be gained in such studies, use of task-based BOLD fMRI to study brain activation in individuals with cerebrovascular disease is not without controversy. Recalling that BOLD signal changes are influenced by factors such as CBV, CBF, and the cerebral rate of metabolic oxygen consumption, pathological changes in these parameters that affect fMRI signals may be mistaken for changes in neuronal activity. For example, a reduction in baseline CBF, due to chronic hypoperfusion will contribute toward a larger BOLD signal if neurons remain viable and active in the region. Beyond hemodynamic parameters, medications, and various other stimulants are known to influence the BOLD-fMRI signal (Pattinson et al., 2009). It is also possible that the temporal characteristics of the BOLD response can be affected after stroke, with evidence for this that dates back a decade (Carusone et al., 2002). Patients with significant extracranial atherosclerotic stenoses or occlusions may have a delayed, abnormal BOLD signal response relative to controls (Donahue et al., 2010), due to potential factors such as collateral vessels or vascular steal. It may be possible to overcome some of the limitations of BOLD fMRI in these cases by performing additional fMRI experiments that rely on non-BOLD contrast, such as using ASL-based methods for CBF-fMRI (see below) or vascular space occupancy (VASO) CBV-fMRI (Kleim et al., 2002). This is an area of functional imaging that needs to be developed if we are to improve our understanding of recovery after stroke.

Another challenging issue associated with task-based BOLD-fMRI relates to observances of absent BOLD signals despite evidence of existing neuronal activity recorded by an alternative functional neuroimaging modality. Others have found in stroke that the amplitude of the motor-related BOLD response is correlated with the capacity of the vessels to dilate, that is the cerebrovascular reactivity (Krainik et al., 2005). Rossini et al. (2004) used BOLD fMRI, magnetoencephalography (MEG), and transcranial doppler (TCD) ultrasound to study chronic stroke survivors and found that although MEG-evoked responses to somatosensory stimuli were well detected, an equivalent BOLD signal was not seen in a subset of patients. In these patients, the absence of task-related BOLD signal was suggested to arise from impaired autoregulation of cerebral perfusion, secondary to cerebrovascular disease. These studies argue for the importance of understanding the underlying vascular health of patients when conducting task-related BOLD fMRI, and the need to confirm findings through use of multiple functional neuroimaging modalities.

### Resting-state BOLD fMRI

Resting-state fMRI (rs-fMRI) is a new branch of human brain mapping that has emerged in recent years (American College of Sports Medicine et al., 2010), although the seminal work occurred in 1995 (Jezzard and Balaban, 1995). A major advantage of rs-fMRI is that it does not require the patient to perform a behavioral task as part of the examination. Thus, concerns regarding abnormal behavioral performance of patients are removed. Brain activity in the resting state is estimated not by task performance, but by low frequency synchrony in the baseline fMRI signal between different brain regions, usually in the frequency range between 0.008 and 0.08 Hz. Maps of spatial synchrony can be obtained by “seed-voxel” approaches, in which the strength of correlations are reported between the signal from one voxel of interest (e.g., in primary motor cortex) and the signal from all other voxels; or by multivariate approaches such as independent component analysis (ICA) (Beckmann et al., 2005). In either the seed voxel or multivariate approaches, the resultant large-scale brain networks are called resting-state networks (RSNs), which are thought to reflect not anatomical connectivity but instead “functional connectivity” of the brain (shown in Figure 3D).

Task-based fMRI has been used for stroke recovery research, while rs-fMRI studies have historically have been less common. This trend appears to be changing, particularly as rs-fMRI can be conducted for patients with profound impairments, rs-fMRI analysis methods have become more widely available (Filippini et al., 2009) and test-retest reliability has been established (Ogoh and Ainslie, 2009). One new phenomenon that would have implications in stroke rs-fMRI research is how reproducible the signals are as a function of time, referred to as the temporal stationarity (Willie and Ainslie, 2011). In the future, it may be possible to find synergies between task and rs-fMRI approaches to advance our understanding of activation patterns that explain or predict recovery after stroke.

### Arterial spin labeling MRI

Another MRI technique for assessing brain function and physiology is ASL. Although discovered and developed contemporaneously, ASL is a complex imaging experiment that historically has not been used as widely as BOLD fMRI (Williams et al., 1992). Recent technical advances have made ASL more popular. Two images are required in ASL; in the first image, RF pulses are used to invert magnetization in arterial blood proximal to the imaging region of interest. The “tagged” or “labeled” blood water subsequently is allowed to flow into the imaging region for a sufficient time to reach the microvasculature, causing a small decrease in the measured MRI signal intensity in proportion to the T1 relaxation properties of the tagged blood in the microvasculature, and the microvasculature volume fraction within a given imaging voxel. The second image (the “control”) has no effective labeling but otherwise near identical MRI signal contrast weighting. The difference between the control and tag images provides an image that is CBF-weighted. Although there are several parameters that must be measured, or more typically assumed, absolute quantification in ASL is relatively straight forward (Wong, 2005; van Osch et al., 2009). The ASL signal difference (i.e., control – tag images) divided by the initial magnetization (i.e., related to the proton density weighting) is directly proportional to perfusion in units of mL/100 g/min.

As for BOLD fMRI, the ASL perfusion signal is limited by the small volume fraction of microvascular blood in tissue (1–2% of the available signal in gray matter). Low SNR is a limitation of ASL and good temporal image stability is required, as many images are typically acquired in a time series and then averaged together to yield a perfusion image. Refinements in MRI hardware and acquisition methods have improved ASL image quality due to: (1) improved gradient technology for fast imaging; (2) availability of higher main magnetic field MR systems (e.g., ≥3 Tesla) (Petersen et al., 2010) that lengthen T1 relaxation times and enhance labeling efficiency; (3) multi-slice capabilities (Gunther et al., 2005) that improve the volume of coverage; and (4) improved theoretical models from which quantitative estimates of CBF can be derived (Chappell et al., 2010).

The ASL technique can be designed to map brain activity analogous to BOLD signal contrast (Aguirre et al., 2002), or to produce baseline CBF images (Alsop and Detre, 1996) as an alternative to PWI, avoiding the use of Gadolinium contrast agents. Two possible advantages of ASL over BOLD-fMRI are that (1) the ASL signal is closely associated with CBF, whereas the BOLD signal is of much more complex signal origins and; (2) the hemodynamic response of the ASL signal to neuronal activity has improved spatial resolution. The reduced SNR of ASL images in relation to BOLD-fMRI images remains a challenge, but the use of ASL to focus on the CBF characteristics of normal and abnormal cerebrovasculature is very promising.

The role that ASL can play in imaging acute and chronic stroke patients remains open for exploration, with the first clinical ASL stroke study dates back over a decade (Chalela et al., 2000). One important consideration is the potential use of ASL instead of PWI, particularly for patients in which Gd-DTPA is contraindicated. Thorough comparisons of the two imaging approaches are now being conducted in the literature (Siewert et al., 1997; Weber et al., 2003; Zaharchuk et al., 2009) and in diagnostic imaging departments worldwide, as MRI vendors have recently made ASL sequences commercially available. PWI and ASL show similar results in healthy brain tissue, but some divergence has been noted in cerebrovascular diseases. One option that appears clinically feasible is the inclusion of a 4 to 6-min ASL acquisition to improve the quantitative accuracy of the PWI-derived CBF maps (Debette and Markus, 2010). Interestingly, among individuals with suspected cerebrovascular disease, roughly half of the patients had an abnormal ASL CBF image while the DSC image was viewed as normal (Zaharchuk et al., 2009).

The ASL method also appears to have an experimental role in assessing minor stroke or chronic stroke recovery. Specifically, it is important to identify whether there are delays in the time it takes the tagged bolus to travel from the labeling plane to the imaging plane. This ASL transit-time metric has an analogous PWI counterpart, and has been referred to as bolus arrival time, the arterial arrival time (MacIntosh et al., 2010) and, perhaps most commonly, the arterial transit-time (ATT or t_A_) (Wang et al., 2003). Vascular pathologies and different vascular status can contribute to a delay or hastening of ASL bolus. If unaccounted for, the delay will manifest as underestimated CBF; decreased ATT will have the opposite effect. Some clinical ASL studies have incorporated ATT estimates, although longer scan durations are often required. Nonetheless, ATT information may be useful in stroke diagnosis (Figures 2C,D, patient 2). Future studies should evaluate how ATT can be used to improve understanding of stroke outcomes.

A recent study used ASL to investigate physiological correlates of dementia at 6 years post-stroke, compared to adults with Alzheimer’s Disease (AD) and healthy controls (Kuller et al., 2004). In this study structural hippocampal volume was the best predictor of AD, whereas the ASL was the best predictor of the post-stroke adults going on to develop dementia. One methodological aspect of this study was the use of a normalization procedure – the global gray to white matter CBF ratio – to increase detection sensitivity as is relatively common in SPECT and PET studies. The normalization of ASL data to provide semi-quantitative images may be a better alternative to absolute CBF images in clinical scenarios, in which vastly different resting CBF levels are observed across patients. Others have shown that use of the normalized CBF image had higher clinical sensitivity in a study of AD patients when compared to using the absolute CBF images (Benar et al., 2002).

### Future directions for MRI research in stroke recovery

Given the breadth and versatility of MRI methodology, there are numerous interesting research directions that can be undertaken to advance the scientific understanding of stroke recovery, ultimately toward improving health care management of chronic stroke patients. In this closing section, three MRI approaches are discussed briefly.

First, similar to the multifaceted acute MRI protocol, the scientific field would benefit from improved integration of multi-parametric MRI protocols for sub-acute and chronic stroke. For example, to date there are few examples integrating DTI white matter tract data, rs-fMRI functional connectivity data, and task-based fMRI data in stroke patients, yet there should be linkages between these datasets. It will be important to assess the extent that each MRI pulse sequence provides unique, clinically relevant information; whether all experiments are required in clinical research protocols; or whether only the most sensitive indicators are practical for expediency. An additional area requiring expanded multi-parametric MRI involves improving the quantitative interpretation of task-based BOLD-fMRI data. With the potential to develop MRI experiments sensitive to parameters such as microvascular CBV and hematocrit, it may be possible to combine such measures with ASL-derived CBF to model the relationship between BOLD-fMRI signal changes and cerebral metabolic rate of oxygen consumption (CMRO2) (Hoge et al., 1999). The prospect of non-invasive CMRO2 maps would provide a good marker of neuronal pathology for stroke patients. Again, the ability to perform such multi-parametric assessments in a timely fashion remains a challenge. However, this is quite important given the heterogeneity of MRI findings across stroke patients, and the wealth of BOLD-fMRI data already collected in stroke patients that would benefit from more detailed evaluation and insight. BOLD-fMRI studies in stroke patients also require additional validation through supplemental measurements using modalities that do not rely on the neurovascular coupling phenomenon. For assessing cortical activity, electroencephalography is relatively low-cost and widely available. As in the case of fMRI, however, electroencephalography datasets are complex and require careful interpretation.

Second, MRI promises to play a role in the interplay between genetics and stroke recovery. Recently, a number of fMRI studies have been undertaken to assess the effects of various genetic polymorphisms on brain activation patterns. As the role of various genetic factors becomes better understood, it is possible that genetic profile may provide some insight into the potential for recovery after stroke, and that assists selection of the appropriate rehabilitation therapy for specific patients (Pearson-Fuhrhop and Cramer, 2010). For example, polymorphisms of the apolipoprotein E (APOE) and brain-derived neurotrophic factor (BDNF) genes may influence outcome after stroke (Cramer and Procaccio, 2012).

A last area that deserves brief mention is the use of MRI to guide intervention and rehabilitation. Technology is continually (albeit slowly) developing toward expanding the list of options for therapeutic intervention for stroke patients in the acute phase. One example of emerging technology involves use of focused ultrasound. Through use of ultrasound transducer arrays with high channel count, it is becoming possible to distribute heat load across the skull while adjusting the transmit phase of individual elements such that they constructively interfere to generate substantial focused ultrasound pressure fields within the brain (Clement and Hynynen, 2002). Such systems require detailed measurements of skull thickness (as provided by CT, for example) to adjust transducer phase appropriately, and MRI guidance to assess the effects of focused ultrasound treatment. MRI guidance is critical not only to detect the effects of ultrasound on anatomical structures, but also because various MRI parameters (and the Larmor frequency, in particular) are temperature sensitive (Peters et al., 1998). Thus, specialized MRI techniques provide a method of non-invasive thermometry that can be used to control focused ultrasound heat treatments. Completely non-invasive thermal ablation treatments are possible, as demonstrated in patients with brain tumors (McDannold et al., 2010), although future applications to stroke are possible. One such approach involves use of MRI guided focused ultrasound not for thermal therapy, but for transient disruption of the blood brain barrier that enables targeted delivery of stem cells for neural repair (Burgess et al., 2011). Another involves “sonothrombolysis,” in which the combination of MRI guided focused ultrasound and injected microbubble agents are potentially used to enhance the ability of tPA to recanalize vessels (Ricci et al., 2012).

To conclude, MRI is a highly versatile imaging modality that can be used in numerous different applications to probe the anatomy and physiology of the healthy and injured brain. Substantial capacity remains for new technical innovation to expand the wealth of biophysical information that MRI provides. In the coming decades, it is very likely that MRI techniques will play a critical role in the development and subsequent clinical implementation of new therapeutic strategies to improve outcome after stroke.

## Conflict of Interest Statement

The authors declare that the research was conducted in the absence of any commercial or financial relationships that could be construed as a potential conflict of interest.

## References

[B1] Adluru N. Zhang H. Fox A. S. Shelton S. E. Ennis C. M. Bartosic A. M. (2012). A diffusion tensor brain template for rhesus macaques. Neuroimage 59, 306–318 10.1016/j.neuroimage.2011.07.029 21803162 PMC3195880

[B2] Aguirre G. K. Detre J. A. Zarahn E. Alsop D. C. (2002). Experimental design and the relative sensitivity of BOLD and perfusion fMRI. Neuroimage 15, 488–500 10.1006/nimg.2001.0990 11848692

[B3] Alexander L. D. Black S. E. Patterson K. K. Gao F. Danells C. J. McIlroy W. E. (2009). Association between gait asymmetry and brain lesion location in stroke patients. Stroke 40, 537–544 10.1161/STROKEAHA.108.527374 19109546

[B4] Alsop D. C. Detre J. A. (1996). Reduced transit-time sensitivity in noninvasive magnetic resonance imaging of human cerebral blood flow. J. Cereb. Blood Flow Metab. 16, 1236–1249 10.1097/00004647-199611000-00019 8898697

[B5] American College of Sports Medicine Thompson W. R. Gordon N. F. Pescatello L. S. (2010). ACSM’s Guidelines for Exercise Testing and Prescription. Philadelphia: Lippincott Williams & Wilkins/Wolters Kluwer

[B6] Ashburner J. Friston K. J. (2000). Voxel-based morphometry – the methods. Neuroimage 11, 805–821 10.1016/S1053-8119(00)91396-X 10860804

[B7] Baird T. A. Parsons M. W. Barber P. A. Butcher K. S. Desmond P. M. Tress B. M. (2002). The influence of diabetes mellitus and hyperglycaemia on stroke incidence and outcome. J. Clin. Neurosci. 9, 618–626 10.1054/jocn.2002.1081 12604269

[B8] Barnes S. R. Haacke E. M. (2009). Susceptibility-weighted imaging: clinical angiographic applications. Magn. Reson. Imaging Clin. N. Am. 17, 47–61 10.1016/j.mric.2008.12.002 19364599 PMC2713115

[B9] Beckmann C. F. DeLuca M. Devlin J. T. Smith S. M. (2005). Investigations into resting-state connectivity using independent component analysis. Philos. Trans. R. Soc. Lond. B Biol. Sci. 360, 1001–1013 10.1098/rstb.2005.1634 16087444 PMC1854918

[B10] Benar C. G. Gross D. W. Wang Y. Petre V. Pike B. Dubeau F. (2002). The BOLD response to interictal epileptiform discharges. Neuroimage 17, 1182–1192 10.1006/nimg.2002.1164 12414258

[B11] Biernaskie J. Chernenko G. Corbett D. (2004). Efficacy of rehabilitative experience declines with time after focal ischemic brain injury. J. Neurosci. 24, 1245–1254 10.1523/JNEUROSCI.3834-03.2004 14762143 PMC6793570

[B12] Bloch F. (1946). Nuclear induction. Phys. Rev. 70, 460–473 10.1103/PhysRev.70.460

[B13] Borresen J. Lambert M. I. (2008). Autonomic control of heart rate during and after exercise: measurements and implications for monitoring training status. Sports Med. 38, 633–646 10.2165/00007256-200838080-00002 18620464

[B14] Broderick J. Phillips S. Whisnant J. O’Fallen W. Bergstralh E. (1989). Incident rates of stroke in the eighties: the end of the decline in stroke? Stroke 20, 577–582 10.1161/01.STR.20.5.577 2718196

[B15] Broderick J. P. William M. (2004). Feinberg lecture: stroke therapy in the year 2025: burden, breakthroughs, and barriers to progress. Stroke 35, 205–211 10.1161/01.STR.0000106160.34316.19 14671248

[B16] Bronskill M. J. Graham S. J. (1993). “NMR characteristics of tissue,” in The Physics of MRI: 1992 AAPM Summer School Proceedings, eds Bronskill M. J. Sprawls P. (Woodbury: American Institute of Physics), 32–55

[B17] Bulte D. P. Kelly M. Germuska M. Xie J. Chappell M. A. Okell T. W. (2012). Quantitative measurement of cerebral physiology using respiratory-calibrated MRI. Neuroimage 60, 582–591 10.1016/j.neuroimage.2011.12.017 22209811 PMC7100043

[B18] Burgess A. Ayala-Grosso C. A. Ganguly M. Jordao J. F. Aubert I. Hynynen K. (2011). Targeted delivery of neural stem cells to the brain using MRI-guided focused ultrasound to disrupt the blood-brain barrier. PLoS ONE 6:e27877 10.1371/journal.pone.0027877 22114718 PMC3218061

[B19] Calautti C. Baron J. C. (2003). Functional neuroimaging studies of motor recovery after stroke in adults: a review. Stroke 34, 1553–1566 10.1161/01.STR.0000071761.36075.A6 12738893

[B20] Carr H. Y. Purcell E. M. (1954). Effects of diffusion on free precession in nuclear magnetic resonance experiments. Phys. Rev. 94, 630–638 10.1103/PhysRev.94.630

[B21] Carusone L. M. Srinivasan J. Gitelman D. R. Mesulam M. M. Parrish T. B. (2002). Hemodynamic response changes in cerebrovascular disease: implications for functional MR imaging. AJNR Am. J. Neuroradiol. 23, 1222–1228 12169483 PMC8185726

[B22] Chalela J. A. Alsop D. C. Gonzalez-Atavales J. B. Maldjian J. A. Kasner S. E. Detre J. A. (2000). Magnetic resonance perfusion imaging in acute ischemic stroke using continuous arterial spin labeling. Stroke 31, 680–687 10.1161/01.STR.31.3.680 10700504

[B23] Chalela J. A. Kidwell C. S. Nentwich L. M. Luby M. Butman J. A. Demchuk A. M. (2007). Magnetic resonance imaging and computed tomography in emergency assessment of patients with suspected acute stroke: a prospective comparison. Lancet 369, 293–298 10.1016/S0140-6736(07)60151-2 17258669 PMC1859855

[B24] Chappell M. A. MacIntosh B. J. Donahue M. J. Günther M. Jezzard P. Woolrich M. W. (2010). Separation of intravascular signal in multi-inversion time arterial spin labelling MRI. Magn. Reson. Med. 63, 1357–1365 10.1002/mrm.22320 20432306

[B25] Cheng B. Brinkmann M. Forkert N. D. Treszl A. Ebinger M. Kohrmann M. (2013). Quantitative measurements of relative fluid-attenuated inversion recovery (FLAIR) signal intensities in acute stroke for the prediction of time from symptom onset. J. Cereb. Blood Flow Metab. 33, 76–84 10.1038/jcbfm.2012.129 23047272 PMC3965287

[B26] Clement G. T. Hynynen K. (2002). A non-invasive method for focusing ultrasound through the human skull. Phys. Med. Biol. 47, 1219–1236 10.1088/0031-9155/47/8/301 12030552

[B27] Cohen M. S. (1997). Parametric analysis of fMRI data using linear systems methods. Neuroimage 6, 93–103 10.1006/nimg.1997.0278 9299383

[B28] Cramer S. C. Nelles G. Benson R. R. Kaplan J. D. Parker R. A. Kwong K. K. (1997). A functional MRI study of subjects recovered from hemiparetic stroke. Stroke 28, 2518–2527 10.1161/01.STR.28.12.2518 9412643

[B29] Cramer S. C. Procaccio V. (2012). Correlation between genetic polymorphisms and stroke recovery: analysis of the GAIN Americas and GAIN International studies. Eur. J. Neurol. 19, 718–724 10.1111/j.1468-1331.2011.03615.x 22221491

[B30] Damadian R. (1971). Tumor detection by nuclear magnetic resonance. Science 171, 1151–1153 10.1126/science.171.3976.1151 5544870

[B31] Davis W. L. Warnock S. H. Harnsberger H. R. Parker D. L. Chen C. X. (1993). Intracranial MRA: single volume vs. multiple thin slab 3D time-of-flight acquisition. J. Comput. Assist. Tomogr. 17, 15–21 10.1097/00004728-199301000-00002 8419427

[B32] De Coene B. Hajnal J. V. Gatehouse P. Longmore D. B. White S. J. Oatridge A. (1992). MR of the brain using fluid-attenuated inversion recovery (FLAIR) pulse sequences. AJNR Am. J. Neuroradiol. 13, 1555–1564 1332459 PMC8332405

[B33] Debette S. Markus H. S. (2010). The clinical importance of white matter hyperintensities on brain magnetic resonance imaging: systematic review and meta-analysis. BMJ 341, c3666 10.1136/bmj.c3666 20660506 PMC2910261

[B34] Donahue M. J. Sideso E. MacIntosh B. J. Kennedy J. Handa A. Jezzard P. (2010). Absolute arterial cerebral blood volume quantification using inflow vascular-space-occupancy with dynamic subtraction magnetic resonance imaging. J. Cereb. Blood Flow Metab. 30, 1329–1342 10.1038/jcbfm.2010.16 20145656 PMC2949227

[B35] Donnan G. A. Baron J. C. Ma H. Davis S. M. (2009). Penumbral selection of patients for trials of acute stroke therapy. Lancet Neurol. 8, 261–269 10.1016/S1474-4422(09)70041-9 19233036

[B36] Fazekas F. Kleinert R. Roob G. Kleinert G. Kapeller P. Schmidt R. (1999). Histopathologic analysis of foci of signal loss on gradient-echo T2*-weighted MR images in patients with spontaneous intracerebral hemorrhage: evidence of microangiopathy-related microbleeds. AJNR Am. J. Neuroradiol. 20, 637–642 10319975 PMC7056037

[B37] Filippini N. MacIntosh B. J. Hough M. G. Goodwin G. M. Frisoni G. B. Smith S. M. (2009). Distinct patterns of brain activity in young carriers of the APOE-{varepsilon}4 allele. Proc. Natl. Acad. Sci. U.S.A. 106, 7209–7214 10.1073/pnas.0811879106 19357304 PMC2678478

[B38] Fischl B. Dale A. M. (2000). Measuring the thickness of the human cerebral cortex from magnetic resonance images. Proc. Natl. Acad. Sci. U.S.A. 97, 11050–11055 10.1073/pnas.200033797 10984517 PMC27146

[B39] Gati J. S. Menon R. S. Ugurbil K. Rutt B. K. (1997). Experimental determination of the BOLD field strength dependence in vessels and tissue. Magn. Reson. Med. 38, 296–302 10.1002/mrm.1910380220 9256111

[B40] Gunther M. Oshio K. Feinberg D. A. (2005). Single-shot 3D imaging techniques improve arterial spin labeling perfusion measurements. Magn. Reson. Med. 54, 491–498 10.1002/mrm.20580 16032686

[B41] Henkelman R. M. Stanisz G. J. Kim J. K. Bronskill M. J. (1994). Anisotropy of NMR properties of tissues. Magn. Reson. Med. 31, 592–601 10.1002/mrm.1910320508 7808260

[B42] Hoge R. D. Atkinson J. Gill B. Crelier G. R. Marrett S. Pike G. B. (1999). Linear coupling between cerebral blood flow and oxygen consumption in activated human cortex. Proc. Natl. Acad. Sci. U.S.A. 96, 9403–9408 10.1073/pnas.96.16.9403 10430955 PMC17795

[B43] Jezzard P. Balaban R. S. (1995). Correction for geometric distortion in echo planar images from B0 field variations. Magn. Reson. Med. 34, 65–73 10.1002/mrm.1910340111 7674900

[B44] Kansagra A. P. Wong E. C. (2008a). Quantitative assessment of mixed cerebral vascular territory supply with vessel encoded arterial spin labeling MRI. Stroke 39, 2980–2985 10.1161/STROKEAHA.108.515767 18703809

[B45] Kansagra A. P. Wong E. C. (2008b). Mapping of vertebral artery perfusion territories using arterial spin labeling MRI. J. Magn. Reson. Imaging 28, 762–766 10.1002/jmri.21462 18777538

[B46] Kidwell C. S. Jahan R. Gornbein J. Alger J. R. Nenov V. Ajani Z. (2013). A trial of imaging selection and endovascular treatment for ischemic stroke. N. Engl. J. Med. 368, 914–923 10.1056/NEJMoa1212793 23394476 PMC3690785

[B47] Kleim J. A. Cooper N. R. VandenBerg P. M. (2002). Exercise induces angiogenesis but does not alter movement representations within rat motor cortex. Brain Res. 934, 1–6 10.1016/S0006-8993(02)02239-4 11937064

[B48] Krainik A. Hund-Georgiadis M. Zysset S. von Cramon D. Y. (2005). Regional impairment of cerebrovascular reactivity and BOLD signal in adults after stroke. Stroke 36, 1146–1152 10.1161/01.STR.0000166178.40973.a7 15879326

[B49] Kruger G. Kastrup A. Glover G. H. (2001). Neuroimaging at 1.5 T and 3.0 T: comparison of oxygenation-sensitive magnetic resonance imaging. Magn. Reson. Med. 45, 595–604 10.1002/mrm.1081 11283987

[B50] Kuller L. H. Longstreth W. T. Jr. Arnold A. M. Bernick C. Bryan R. N. Beauchamp N. J. Jr. (2004). White matter hyperintensity on cranial magnetic resonance imaging: a predictor of stroke. Stroke 35, 1821–1825 10.1161/01.STR.0000132193.35955.69 15178824

[B51] Labeyrie M. A. Turc G. Hess A. Hervo P. Mas J. L. Meder J. F. (2012). Diffusion lesion reversal after thrombolysis: a MR correlate of early neurological improvement. Stroke 43, 2986–2991 10.1161/STROKEAHA.112.661009 22996954

[B52] Lauterbur P. C. (1973). Image formation by induced local interactions: examples employing nuclear magnetic resonance. Nature 242, 190–191 10.1038/242190a0 2663289

[B53] Leiva-Salinas C. Wintermark M. Kidwell C. S. (2011). Neuroimaging of cerebral ischemia and infarction. Neurotherapeutics 8, 19–27 10.1007/s13311-010-0004-2 21274682 PMC3075733

[B54] Logothetis N. K. Pauls J. Augath M. Trinath T. Oeltermann A. (2001). Neurophysiological investigation of the basis of the fMRI signal. Nature 412, 150–157 10.1038/35084005 11449264

[B55] Longstreth W. T. Jr. Bernick C. Manolio T. A. Bryan N. Jungreis C. A. Price T. R. (1998). Lacunar infarcts defined by magnetic resonance imaging of 3660 elderly people: the Cardiovascular Health Study. Arch. Neurol. 55, 1217–1225 10.1001/archneur.55.9.1217 9740116

[B56] Lu H. Nagae-Poetscher L. M. Golay X. Lin D. Pomper M. van Zijl P. C. (2005). Routine clinical brain MRI sequences for use at 3.0 Tesla. J. Magn. Reson. Imaging 22, 13–22 10.1002/jmri.20356 15971174

[B57] MacIntosh B. J. Baker S. N. Mraz R. Ives J. R. Martel A. L. McIlroy W. E. (2007). Improving functional magnetic resonance imaging motor studies through simultaneous electromyography recordings. Hum. Brain Mapp. 28, 835–845 10.1002/hbm.20308 17133382 PMC4898954

[B58] MacIntosh B. J. Filippini N. Chappell M. A. Woolrich M. W. Mackay C. E. Jezzard P. (2010). Assessment of arterial arrival times derived from multiple inversion time pulsed arterial spin labeling MRI. Magn. Reson. Med. 63, 641–647 10.1002/mrm.22256 20146233

[B59] MacIntosh B. J. McIlroy W. E. Mraz R. Staines W. R. Black S. E. Graham S. J. (2008). Electrodermal recording and fMRI to inform sensorimotor recovery in stroke patients. Neurorehabil. Neural Repair 22, 728–736 10.1177/1545968308316386 18784267 PMC4896813

[B60] Mascalchi M. Filippi M. Floris R. Fonda C. Gasparotti R. Villari N. (2005). Diffusion-weighted MR of the brain: methodology and clinical application. Radiol. Med. 109, 155–197 15775887

[B61] McDannold N. Clement G. T. Black P. Jolesz F. Hynynen K. (2010). Transcranial magnetic resonance imaging-guided focused ultrasound surgery of brain tumors: initial findings in 3 patients. Neurosurgery 66, 323–332 (discussion 332) 10.1227/01.NEU.0000360379.95800.2F 20087132 PMC2939497

[B62] Menon R. S. Goodyear B. G. (1999). Submillimetre functional localization in human striate cortex using BOLD contrast at 4 Tesla: implications for the vascular point-spread function. Magn. Reson. Med. 41, 230–235 10.1002/(SICI)1522-2594(199902)41:2<230::AID-MRM3>3.0.CO;2-O 10080267

[B63] Morita S. Masukawa A. Suzuki K. Hirata M. Kojima S. Ueno E. (2011). Unenhanced MR angiography: techniques and clinical applications in patients with chronic kidney disease. Radiographics 31, E13–E33 10.1148/rg.312105075 21415179

[B64] Nencka A. S. Rowe D. B. (2007). Reducing the unwanted draining vein BOLD contribution in fMRI with statistical post-processing methods. Neuroimage 37, 177–188 10.1016/j.neuroimage.2007.03.075 17560130

[B65] Norris D. G. (2006). Principles of magnetic resonance assessment of brain function. J. Magn. Reson. Imaging 23, 794–807 10.1002/jmri.20587 16649206

[B66] Nucifora P. G. Verma R. Lee S. K. Melhem E. R. (2007). Diffusion-tensor MR imaging and tractography: exploring brain microstructure and connectivity. Radiology 245, 367–384 10.1148/radiol.2452060445 17940300

[B67] Ogawa S. Lee T. M. Kay A. R. Tank D. W. (1990). Brain magnetic resonance imaging with contrast dependent on blood oxygenation. Proc. Natl. Acad. Sci. U.S.A. 87, 9868–9872 10.1073/pnas.87.24.9868 2124706 PMC55275

[B68] Ogoh S. Ainslie P. N. (2009). Cerebral blood flow during exercise: mechanisms of regulation. J. Appl. Physiol. 107, 1370–1380 10.1152/japplphysiol.00573.2009 19729591

[B69] Pattinson K. T. Governo R. J. MacIntosh B. J. Russell E. C. Corfield D. R. Tracey I. (2009). Opioids depress cortical centers responsible for the volitional control of respiration. J. Neurosci. 29, 8177–8186 10.1523/JNEUROSCI.1375-09.2009 19553457 PMC6666048

[B70] Pearson-Fuhrhop K. M. Cramer S. C. (2010). Genetic influences on neural plasticity. PM R 2, S227–40 10.1016/j.pmrj.2010.09.011 21172685

[B71] Peters R. D. Hinks R. S. Henkelman R. M. (1998). Ex vivo tissue-type invariability in proton-resonance frequency shift MR thermometry. Magn. Reson. Med. 40, 454–459 10.1002/mrm.1910400316 9727949

[B72] Petersen E. T. Mouridsen K. Golay X. (2010). The QUASAR reproducibility study, part II: results from a multi-center arterial spin labeling test-retest study. Neuroimage 49, 104–113 10.1016/j.neuroimage.2009.07.068 19660557 PMC2768325

[B73] Price C. J. Crinion J. Friston K. J. (2006). Design and analysis of fMRI studies with neurologically impaired patients. J. Magn. Reson. Imaging 23, 816–826 10.1002/jmri.20580 16649208

[B74] Purcell E. M. Torrey H. C. Pound R. V. (1946). Resonance absorption by nuclear magnetic moments in a solid. Phys. Rev. 69, 37–38 10.1103/PhysRev.69.37

[B75] Radlinska B. Ghinani S. I. Leppert R. Minuk J. Pike G. B. Thiel A. (2010). Diffusion tensor imaging, permanent pyramidal tract damage, and outcome in subcortical stroke. Neurology 75, 1048–1054 10.1212/WNL.0b013e3181f39aa0 20855848 PMC2942063

[B76] Ricci S. Dinia L. Del Sette M. Anzola P. Mazzoli T. Cenciarelli S. (2012). Sonothrombolysis for acute ischaemic stroke. Cochrane Database Syst. Rev. 6, CD008348 22696378 10.1002/14651858.CD008348.pub2

[B77] Rossini P. M. Altamura C. Ferretti A. Vernieri F. Zappasodi F. Caulo M. (2004). Does cerebrovascular disease affect the coupling between neuronal activity and local haemodynamics? Brain 127, 99–110 10.1093/brain/awh012 14570819

[B78] Rotenberg D. Chiew M. Ranieri S. Tam F. Chopra R. Graham S. J. (2013). Real-time correction by optical tracking with integrated geometric distortion correction for reducing motion artifacts in functional MRI. Magn. Reson. Med. 69, 734–748 10.1002/mrm.24309 22585554

[B79] Runge V. M. Kirsch J. E. Lee C. (1993). Contrast-enhanced MR angiography. J. Magn. Reson. Imaging 3, 233–239 10.1002/jmri.1880030135 8428091

[B80] Shelton F. N. Reding M. J. (2001). Effect of lesion location on upper limb motor recovery after stroke. Stroke 32, 107–112 10.1161/01.STR.32.1.107 11136923

[B81] Siewert B. Schlaug G. Edelman R. R. Warach S. (1997). Comparison of EPISTAR and T2*-weighted gadolinium-enhanced perfusion imaging in patients with acute cerebral ischemia. Neurology 48, 673–679 10.1212/WNL.48.3.673 9065546

[B82] Smith J. C. Paulson E. S. Cook D. B. Verber M. D. Tian Q. (2010). Detecting changes in human cerebral blood flow after acute exercise using arterial spin labeling: implications for fMRI. J. Neurosci. Methods 191, 258–262 10.1016/j.jneumeth.2010.06.028 20603148

[B83] Sohn C. H. Sevick R. J. Frayne R. (2003). Contrast-enhanced MR angiography of the intracranial circulation. Magn. Reson. Imaging Clin. N. Am. 11, 599–614 10.1016/S1064-9689(03)00064-3 15018113

[B84] Stehling M. K. Turner R. Mansfield P. (1991). Echo-planar imaging: magnetic resonance imaging in a fraction of a second. Science 254, 43–50 10.1126/science.1925560 1925560

[B85] Taussky P. Tawk R. G. Daugherty W. P. Hanel R. A. (2011). Medical therapy for ischemic stroke: review of intravenous and intra-arterial treatment options. World Neurosurg. 76, S9–15 10.1016/j.wneu.2011.05.048 22182278

[B86] Tenser M. S. Amar A. P. Mack W. J. (2011). Mechanical thrombectomy for acute ischemic stroke using the MERCI retriever and penumbra aspiration systems. World Neurosurg. 76, S16–23 10.1016/j.wneu.2011.07.003 22182267

[B87] Thomalla G. Rossbach P. Rosenkranz M. Siemonsen S. Krutzelmann A. Fiehler J. (2009). Negative fluid-attenuated inversion recovery imaging identifies acute ischemic stroke at 3 hours or less. Ann. Neurol. 65, 724–732 10.1002/ana.21651 19557859

[B88] Thompson C. S. Hakim A. M. (2009). Living beyond our physiological means: small vessel disease of the brain is an expression of a systemic failure in arteriolar function: a unifying hypothesis. Stroke 40, e322–e330 10.1161/STROKEAHA.108.542266 19228835

[B89] Van Essen D. C. Ugurbil K. Auerbach E. Barch D. Behrens T. E. Bucholz R. (2012). The Human Connectome Project: a data acquisition perspective. Neuroimage 62, 2222–2231 10.1016/j.neuroimage.2012.02.018 22366334 PMC3606888

[B90] van Osch M. J. Teeuwisse W. M. Walderveen M. A. A. Hendrikse J. Kies D. A. van Buchem M. A. (2009). Can arterial spin labeling detect white matter perfusion signal? Magn. Reson. Med. 62, 165–173 10.1002/mrm.22002 19365865

[B91] Wang J. Alsop D. C. Song H. K. Maldjian J. A. Tang K. Salvucci A. E. (2003). Arterial transit time imaging with flow encoding arterial spin tagging (FEAST). Magn. Reson. Med. 50, 599–607 10.1002/mrm.10559 12939768

[B92] Wasserman B. A. (2010). Advanced contrast-enhanced MRI for looking beyond the lumen to predict stroke: building a risk profile for carotid plaque. Stroke 41, S12–6 10.1161/STROKEAHA.110.596288 20876485

[B93] Weber M. A. Gunther M. Lichy M. P. Delorme S. Bongers A. Thilmann C. (2003). Comparison of arterial spin-labeling techniques and dynamic susceptibility-weighted contrast-enhanced MRI in perfusion imaging of normal brain tissue. Invest. Radiol. 38, 712–718 10.1097/01.rli.0000084890.57197.54 14566181

[B94] Williams D. S. Detre J. A. Leigh J. S. Koretsky A. P. (1992). Magnetic resonance imaging of perfusion using spin inversion of arterial water. Proc. Natl. Acad. Sci. U.S.A. 89, 212–216 10.1073/pnas.89.1.212 1729691 PMC48206

[B95] Willie C. K. Ainslie P. N. (2011). Cool head, hot brain: cerebral blood flow distribution during exercise. J. Physiol. (Lond.) 589, 2657–2658 10.1113/jphysiol.2011.209668 21632524 PMC3112541

[B96] Wong E. C. (2005). Quantifying CBF with pulsed ASL: technical and pulse sequence factors. J. Magn. Reson. Imaging 22, 727–731 10.1002/jmri.20459 16261572

[B97] Wu W. C. Wong E. C. (2006). Intravascular effect in velocity-selective arterial spin labeling: the choice of inflow time and cutoff velocity. Neuroimage 32, 122–128 10.1016/j.neuroimage.2006.03.001 16713716

[B98] Zaharchuk G. Bammer R. Straka M. Shankaranarayan A. Alsop D. C. Fischbein N. J. (2009). Arterial spin-label imaging in patients with normal bolus perfusion-weighted MR imaging findings: pilot identification of the borderzone sign. Radiology 252, 797–807 10.1148/radiol.2523082018 19703858 PMC6939961

[B99] Zwanenburg J. J. Hendrikse J. Luijten P. R. (2012). Generalized multiple-layer appearance of the cerebral cortex with 3D FLAIR 7.0-T MR imaging. Radiology 262, 995–1001 10.1148/radiol.11110812 22357899

